# Structural Characterization, and Antioxidant, Hypoglycemic and Immunomodulatory Activity of Exopolysaccharide from *Sanghuangporus sanghuang* JM-1

**DOI:** 10.3390/molecules29194564

**Published:** 2024-09-25

**Authors:** Yanglan Luo, Naixin Cao, Liling Huang, Lanlan Tang, Xuzhou Liu, Wenlong Zhang, Shilv Huang, Xiuchao Xie, Yong Yan

**Affiliations:** 1 Microbiology Research Institute, Guangxi Academy of Agricultural Sciences, Nanning 530007, China; 18509164645@163.com (Y.L.);; 2 Guangxi Germplasm Resource Bank of National Agricultural Microbial Resource Center, Nanning 530007, China; 3 Shannxi Tanchi Biotech Co., Ltd., Yulin 718411, China; caonx123456@163.com; 4 School of Biological Science and Engineering, Shaanxi University of Technology, Hanzhong 723000, China; 5 Lueyang County Test and Inspection Center for Quality and Safety of Agricultural Products, Hanzhong 724300, China

**Keywords:** *Sanghuangporus sanghuang*, exopolysaccharide, structural characterization, antioxidant, hypoglycemic activity, immunomodulatory activity

## Abstract

Sanghuang as a medicinal fungus in China has a history of more than 2000 years, and is known as the “forest gold”. Most notably, the polysaccharides of *Sanghuangporus* sp. have attracted widespread attention due to their significant bioactivity in recent years. At present, extensive studies are being carried out on the extraction methods, structural characterization, and activity evaluation of polysaccharides. Here, we aimed to evaluate the structure and bioactivity of LEPS-1, an exopolysaccharide derived from the *S. sanghuang* JM-1 strain. The structure was elucidated by chromatography/spectral methods and hydrolyzation, and the solubility, the antioxidant activity, hypoglycemic activity and immunomodulatory activity were investigated. Results showed that LEPS-1 contained a →2)-α-Manp(1→6)-α-Galp(1→[2)-α-Manp(1→]_n_→2,6)-α-Manp(1→6,2)-α-Manp(1→3)-α-Manp(1→ backbone substituted at the *O*-6 and *O*-2 positions with side chains. These two branching fragments were β-Manp(1→. The molecular weight of LEPS-1 is 36.131 kDa. The results of biological activity analysis suggested that LEPS-1 was easily soluble in water, with reducing capability and DPPH radical scavenging capability. Furthermore, the IC_50_ values of LEPS-1 against α-amylase and α-glucosidase were 0.96 mg/mL and 1.92 mg/mL. LEPS-1 stimulated RAW264.7 cells to release NO, TNF-α and IL-6 with no cytotoxicity, showing potent potential for immunomodulatory activity. These findings describe a potential natural exopolysaccharide with medicinal value and a basis for the development of *S. sanghuang* exopolysaccharides.

## 1. Introduction

“Sanghuang”, a perennial medicinal fungus, is widespread in Asia [1], such as in China, Myanmar, Korea and Japan, and mainly includes five genera: *Sanghuangporus*, *Fomitopsis*, *Phellinus*, *Fomitiporia* and *Inonotus*, among which *Sanghuangporus* contains 18 species [2]. *Sanghuangporus sanghuang* (Sheng H. Wu, T. Hatt. & Y. C. Dai) uniquely growing on mulberry trees is the true Sanghuang species [3,4,5]. *S. sanghuang* has very important medicinal value in the field of medicine and health care [6]. Over the past ten years, frontier research has demonstrated a large number of its promising pharmacological and therapeutic benefits, such as anti-inflammatory [7], antioxidant [8], antitumor [9], hypoglycemic [10] and immunomodulatory [11] benefits. *S. sanghuang* has been used in folk medicine and traditional medicine for the treatment of patients with cancer, diarrhea, hemorrhage and some menstrual-related disorders [12]. Various traditional Chinese medicinal books including the “Shen Nong Materia Medica” (Eastern Han Dynasty), “Characters of Drugs” (Tang Dynasty), “Illustrated Classic of Materia Medica” (Song Dynasty) and “Compendium of Materia Medica” (Ming Dynasty) have recorded the medicinal value of *S. sanghuang*. In Korea, both folk medicine books “Hyang YakJipSungBang” and “Donguibogam” call *Phellinus* mushrooms a panacea [13]. The main active components of *S. sanghuang* include polysaccharides (PSs), terpenoids, flavonoids, phenols, amino acids, alkaloids, coumarins, lignanoids and others [1,14], among which PSs, terpenoids and flavonoids are the main components [15]. PSs, as biosynthesized macromolecules, are considered the main biological components, which present in fruiting bodies, mycelium or fermentation broth in *S. sanghuang* [16,17]. According to the polysaccharide source, it can be divided into intracellular polysaccharides (IPSs) and exopolysaccharides (EPSs) [18]. The EPSs are a class of high molecular weight extracellular biopolymers consisting of polymerized monosaccharides [19]. The diverse biological activities displayed by EPSs of *Sanghuangporus* spp. include antitumor, immunomodulatory, anti-inflammatory and antioxidant activity [1,20,21].

Interestingly, EPSs of *S. sanghuang* exist in the form of heteropolysaccharides including glucose, mannose and galactose [22]. For example, the SSEPS2 isolated by Cheng et al. [23] was composed of 1,3-linked and 1,2-linked α-D-mannopyranose (Man*p*), with a substitution at *O*-6 of 1,2-linked α-D-Man*p* by 1,6-linked α-D-Man*p* residues and terminal α-D-Man*p* residues. Ma et al. [24] isolated an EPS SHP-2 with a molecular weight of 160 kDa from the fermentation broth of *S. sanghuang*, with a main chain of →4)-β-Man*p*-(1→4)-α-Ara*f*-(1→3, 4)-α-Glc*p*(1→3, 4)-α-Glc*p*-(1→3, 4)-α-Glc*p*-(1→3, 4)-α-Glc*p*-(1→3, 4)-α-Glc*p*-(1→6)-α-Gal*p*-(1→4)-β-Man*p*-(1→ and five branches, including four α-D-Glc*p*-(1→ and one α-D-Man*p*-(1→. The stability, hydrophilicity and biodegradability, along with other properties like the diversity of the physicochemical properties of natural polysaccharides, provide the basis for its wide range of biological activities [25]. Meanwhile, the wild germplasm resources of *S. sanghuang* are exiguous, and the artificial cultivable germplasm resources cultivated are limited at present. However, the fermentation product of *S. sanghuang*, which is easier to obtain in the industry, attracts increasing attention due to its bioactivities that are similar to the fruiting bodies [26]. Consequently, compared with the IPSs from fruiting bodies and the mycelia of *Sanghuangporus* spp., EPS from fermentation broth is more easily obtained. The production of EPS by submerged fermentation can not only reduce the cost but also realize the enrichment of exopolysaccharides in a short time.

In recent years, many researchers have focused on the classification and identification of species, extraction, isolation and purification techniques, structural characteristics, pharmacological activities and possible mechanisms of polysaccharides from various *Sanghuangporus* spp. species [27,28,29,30]. The monosaccharide composition, molecular weight, solubility, chain conformation and biological activity of *S. sanghuang* exopolysaccharides have not been fully understood, and the research on effective active ingredients and mechanisms of action are still in early stages. Therefore, in order to enrich the types, structure and activity of exopolysaccharides of *S. sanghuang*, this study aimed to purify and characterize the exopolysaccharide of the *S. sanghuang* strain JM-1, and further explored their solubility, antioxidant activity, hypoglycemic activity and immunomodulatory activity, providing certain application values for the development and utilization of *S. sanghuang*.

## 2. Results

### 2.1. Extraction and Purification of EPS

**Figure 1:**
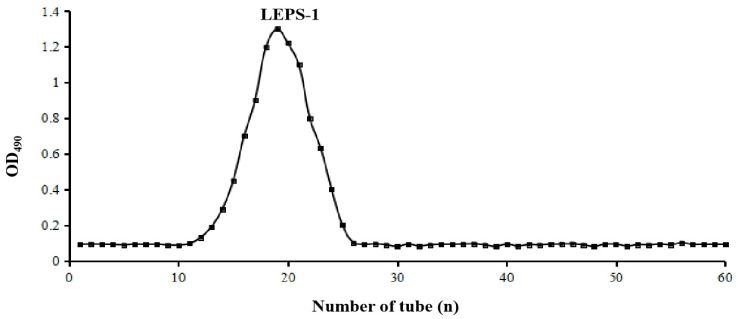
Elution curve of LEPS on Sephadex G-75 gel chromatography column. The LEPS was obtained by dialyzed with a dialysis bag (Mw 3.5kD, MD 55 mm) and lyophilized. The LEPS was further purified by D101 macroporous adsorption resin, DEAE-52 column and Sephadex G-75 gel column to obtain LEPS-1.

The yield of crude polysaccharide EPS was 500 mg/L. After purification with a Sephadex G-75 column, the main component named LEPS-1 was isolated (Figure 1). The contents of total sugars, proteins and uronic acids (such as galacturonic acid and guluronic acid) in LEPS-1 were determined by the phenol–sulfuric acid method, BCA method and 3-hydroxybiphenyl colorimetry, respectively. The total sugar, protein and uronic acid of EPS were 60.65%, 19.68% and 15.27%, respectively. The total sugar and protein of LEPS-1 were 86.48% and 0.39%, respectively. And no glucuronic acid was detected, indicating that LEPS-1 is a neutral sugar.

### 2.2. Chemical Composition of LEPS-1

According to the UV spectrum analysis (Figure S2), it can be seen that the polysaccharide component of LEPS-1 has hardly visible and weak absorption peaks at 260 nm approximately, indicating that there is no nucleic acid in the component. According to the thin chromatographic analysis (Figure S3), the main component of LEPS-1 is mannose.

### 2.3. MW and Monosaccharide Composition of LEPS-1

The molecular weight of polysaccharides is one of the most important factors affecting the expression of their biological activities [31]. The molecular weight of LEPS-1 was 36.131 kDa as determined by HPSEC (Figure S4). According to the ion chromatography in Figure S5A, 13 standard monosaccharides (10 μg/mL) were separated well, including fucose (Fuc), arabinose (Ara), rhamnose (Rha), galactose (Gla), glucose (GlC), xylose (Xyl), mannose (Man), fructose (Fru), ribose (Rib), galacturonic acid (Gal-UA), guluronic acid (Gul-UA), glucuronic acid (Glc-UA) and mannuronic acid (Man-UA). As shown in Figure S5B, LEPS-1 has 8 monosaccharides, namely fucose, arabinose, galactose, glucose, xylose, mannose, galacturonic acid and guluronic acid, in which mannose has the largest peak area, indicating that LEPS-1 is a heteropolysaccharide mainly composed of mannose, consistent with the results of thin layer chromatography (Figure S3). The results suggested that Fuc:Ara:Gal:Glc:Xyl:Man:Gal-UA:Gul-UA = 1.18:2.15:8.16:3.92:0.77:82.38:0.80:0.65. The content of uronic acid in LEPS-1 is relatively low at 1.45%, indicating that LEPS-1 is a neutral sugar.

### 2.4. Characterization of Chemical Structure of LEPS-1

#### 2.4.1. Methylation and Gas Chromatography–Mass Spectrometry Analysis

Based on the characteristic ion fragments in GC-MS (Figure S6), the glycosidic linkage types of LEPS-1 mainly contain 12 glycosidic linkages (Table 1), among which arabinose and fucose have t-Ara (*f*) (1.97%) and t-Fuc (*p*) (0.61%) glycosidic linkages, respectively. Mannose exists in five glycosidic linkages: t-Man (*p*) (35.70%), 3-Man (*p*) (5.07%), 2-Man (*p*) (24.41%), 3,6-Man (*p*) (3.91%) and 2,6-Man (*p*) (0.82%). Glucose exists in two glycosidic linkages: 6-Glc (*p*) (7.17%) and 4-Glc (*p*) (0.82%). Galactose exists in three glycosidic linkages: 6-Gal (*p*) (18.07%), 3,6-Gal (*p*) (0.31%) and 2,6-Gal (*p*) (1.14%).

#### 2.4.2. FT-IR Spectroscopy Measurement

**Figure 2:**
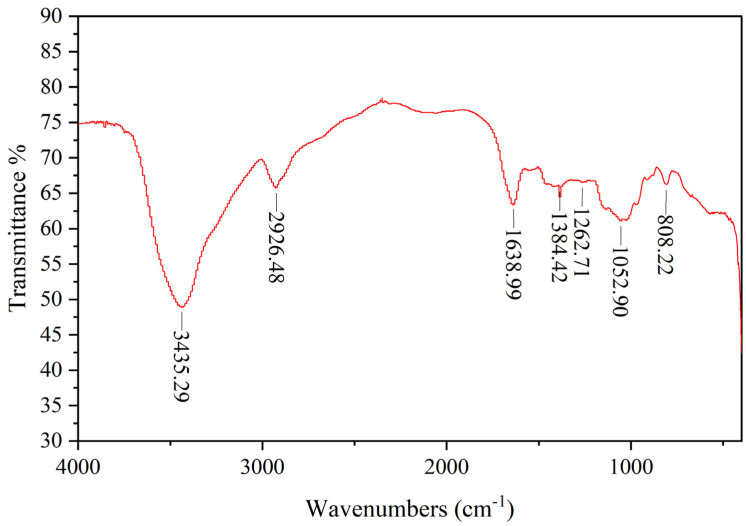
IR spectrum of LEPS-1 in the range of 4000~400 cm^−1^. A broad and strong absorption peak appears at 3435.89 cm^−1^ and was attributed to the stretching vibration of hydroxyl O-H. The strong absorption peak at 2926.48 cm^−1^ was attributed to the stretching vibration of polysaccharide alkyl C-H. The absorption peaks at 1638.99 cm^−1^, 1384.42 cm^−1^ and 1052.90 cm^−1^ were characteristic of C=O stretching vibration and O-H bending vibration, respectively. And the absorption peaks within the range of 1000~700 cm^−1^ were glycosidic bond.

LEPS-1 exhibits characteristic absorption peaks of polysaccharides in the range of 4000–400 cm^−1^ (Figure 2). The absorption peaks that appear in the range of 3600–3200 cm^−1^ were characteristic peaks of sugars, indicating that LEPS-1 contains typical functional groups of polysaccharide substances [32]. A broad and strong absorption peak appears at 3435.89 cm^−1^, indicating the presence of hydrogen bonds between and within the molecules of LEPS-1, which was attributed to the stretching vibration of hydroxyl O-H [33]. The strong absorption peak at 2926.48 cm^−1^ was attributed to the stretching vibration of polysaccharide alkyl C-H, which was also a characteristic peak of carbohydrates [24,34]. No significant peak was detected near 1730 cm^−1^, indicating that the uronic acid in LEPS-1 was not esterified or the absorption peak was masked due to the low content [35]. The absorption peaks at 1638.99 cm^−1^, 1384.42 cm^−1^ and 1052.90 cm^−1^ were characteristic of C=O stretching vibration and O-H bending vibration, respectively. And then the absorption peaks that occur within the range of 1000~700 cm^−1^ were α-configuration glycosidic bond and β-configuration glycosidic bond [36]. All data above are characteristic absorption peaks of sugar.

#### 2.4.3. NMR Analysis

**Figure 3:**
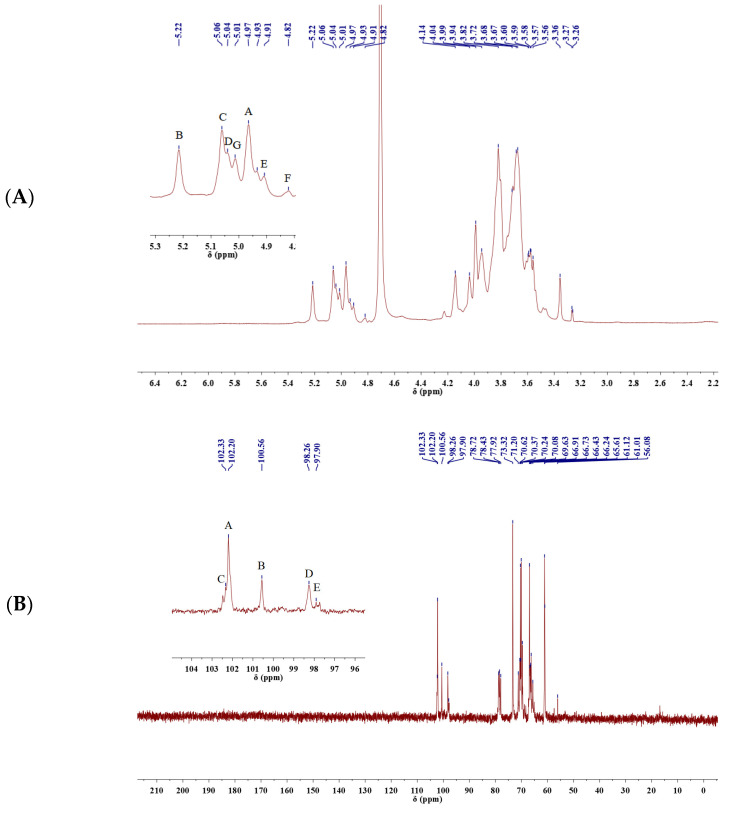
^1^H-NMR (**A**) and ^13^C-NMR (**B**) spectra of LEPS-1. The proton signals were downfield of 4.80 and the coupling constant was 3–4 Hz, indicating that the sugar residues were α-configured. Because signals within 82–88 were not detected, all sugar residues should be in pyranose form.

The signal peaks which mainly appear at δ 3.2~5.4 ppm in the ^1^H NMR spectrum and 60–110 in the ^13^C NMR spectrum (Figure 3) are characteristic of polysaccharides. Seven coupled signal peaks were identified in the heterotopic signal area of δ 4.4~5.3 ppm, indicating that this sample contains seven sugar residues at least, and these were labeled as A, B, C, D, E, F and G (Figure 3A). As the proton signals were downfield of 4.80 and the coupling constant was 3–4 Hz, we inferred that the sugar residues were α-configured [37]. The proton signals of δ 4.4~5.3 ppm belong to C2–C6 [38]. The absence of signals downfield of 170 in the ^13^C NMR spectrum indicated that uronic acid was absent in LEPS-1 (Figure 3B). Because signals within 82–88 were not detected, all sugar residues should be in pyranose form. The ^13^C NMR spectrum showed four signals in the anomeric region at 102.21, 100.57, 102.34, 98.26 and 97.89, which further confirmed that the glycosidic linkages were α-configured. The C2–C6 signals appeared at 62–82 [39]. There is a strong signal peak near δ 102.34 ppm for LEPS-1 (Figure 3B), indicating that the heterocarbon signal of some residues overlap here. Based on the LEPS-1 bonding structure information, heterotopic signals, residue A was β-Man*p*(1→, residue B was →2)-α-Man*p*(1→, residue C was →2,6)-α-Man*p*(1→, residue D was →6)-α-Gal*p*(1→ and residue E was →3)-α-Man*p*(1→. Due to the low content of other sugar residues (F, G, etc.), the signal was weak in HSQC and ^13^C NMR, which requires further analysis.

**Figure 4:**
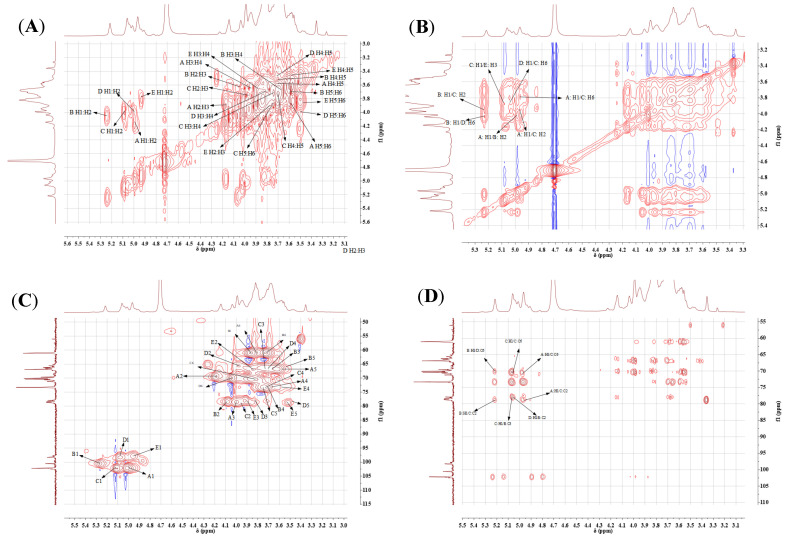
The 2D NMR spectra of LEPS-1. ^1^H-^1^H COSY (**A**), NOESY (**B**), HSQC (**C**) and HMBC (**D**). Residue A was β-Man*p*(1→; residue B was →2)-α-Man*p*(1→; residue C was →2,6)-α-Man*p*(1→; residue D was →6)-α-Gal*p*(1→; and residue E was →3)-α-Man*p*(1→.

**Figure 5:**
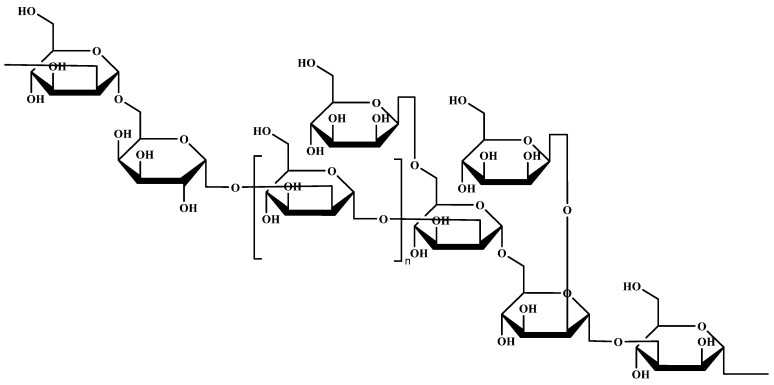
The structure of LEPS-1. Residue A was β-Man*p*(1→; residue B was →2)-α-Man*p*(1→; residue C was →2,6)-α-Man*p*(1→; residue D was →6)-α-Gal*p*(1→; and residue E was →3)-α-Man*p*(1→. The main chain of LEPS-1 mainly consists of →2)-α-Man*p*(1→, →2,6)-α-Man*p*(1→ with a small amount of →6)-α-Gal*p*(1→ and →3)-α-Man*p*(1→. The β-Man*p*(1→ were connected as a terminal sugar at positions 6 and 2 of residue C. Therefore, the structure of LEPS-1 is speculated to be a →2)-α-Manp(1→6)-α-Galp(1→[2)-α-Manp(1→]_n_→2,6)-α-Manp(1→6,2)-α-Manp(1→3)-α-Manp(1→ backbone substituted at the *O*-6 and *O*-2 positions with side chains. These two branching fragments were β-Manp(1→.

The correlation between the proton and carbon signals are confirmed by 2D NMR (Figure 4). The H-1 resonances at 4.97, 5.22, 5.06, 5.02 and 4.91 were assigned to A, B, C, D and E, respectively. According to the HSQC spectrum (Figure 4C), the anomeric carbon signals at 102.21, 100.57, 102.34, 98.26 and 97.89 were correlated with their corresponding proton signals assigned to A, B, C, D and E, respectively. The non-anomeric carbon and proton signals were also identified and were correlated with the chemical shifts shown in Table 2. The HMBC spectrum elucidated the inter-residual coupling signals. There are coupling signals between H-1 (4.97 ppm) of residue A and C-2 (78.76 ppm), C-6 (70.47 ppm) of residue C (A1/C2, A1/C6). There are coupling signals between H-1 (5.22 ppm) of residue B and C-2 (78.76 ppm) of residue C (B1/C2), as well as H-1 (5.22 ppm) of residue B and C-6 (70.18 ppm) of residue D (B1/D6). The coupling signals between H-1 (5.06 ppm) of residue C and C-6 (70.47 ppm) of residue C (C1/C6), H-1 (5.06 ppm) of residue C and C-3 (77.91 ppm) of residue E (C1/E3) as well as H-1 (5.02 ppm) of residue D and C-2 (78.51 ppm) of residue B (D1/B2) were detected. In addition, the NOESY spectrum was conducted to confirm the order of linkages of each residue in the exopolysaccharide. As the NOESY spectrum shows (Figure 4C), the inter-residual cross-peaks were identified between H-1 (4.97 ppm) of residue A and H-2 (4.04 ppm) of residue B (A: H-1/B: H-2), H-1 (4.97 ppm) of residue A and H-2 (3.95 ppm), H-6 (3.82 ppm) of residue C (A: H-1/C: H-2, A: H-1/C: H-6), H-1 (5.22 ppm) of residue B and H-2 (3.95 ppm) of residue C (B: H-1/C: H-2), H-1 (5.22 ppm) of residue B and H-6 (3.99 ppm) of residue D (B: H-1/D: H-6), H-1 (5.06 ppm) of residue C and H-3 (3.87 ppm) of residue E (C: H-1/E: H-3) as well as H-1 (5.02 ppm) of residue D and H-6 (3.87 ppm) of residue C (D: H-1/C: H-6). Combining 1D and 2D NMR information, the main chain of LEPS-1 mainly consists of →2)-α-Man*p*(1→, →2,6)-α-Man*p*(1→ with a small amount of →6)-α-Gal*p*(1→ and →3)-α-Man*p*(1→. The β-Man*p*(1→ were connected as the terminal sugar at positions 6 and 2 of residue C. Therefore, the structure of LEPS-1 is speculated to be a →2)-α-Manp(1→6)-α-Galp(1→[2)-α-Manp(1→]_n_→2,6)-α-Manp(1→6,2)-α-Manp(1→3)-α-Manp(1→ backbone substituted at the *O*-6 and *O*-2 positions with side chains. These two branching fragments were β-Manp(1→. (Figure 5).

### 2.5. Evaluation of Biological Activities

#### 2.5.1. Solubility of LEPS-1

At room temperature, LEPS-1 was easily soluble in water, and micro-soluble in 5% NaOH solution and DMSO. The maximum saturable amount is 46.79 g/L. It was insoluble in organic solvents such as ethanol, methanol, trichloromethane and acetone.

#### 2.5.2. Antioxidant Activity

**Figure 6:**
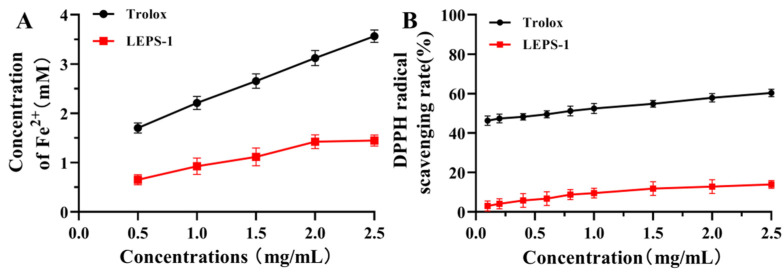
Antioxidant activities of LEPS-1 in vitro. Assay of reducing power (**A**) and DPPH free radical scavenging capability (**B**). The reducing capability of LEPS-1 was lower than that of Trolox in the range of 0.5–2.5 mg/mL. The DPPH radical scavenging capability increased with increased LEPS-1 concentration, and LEPS-1 was 13.95% at 2.5 mg/mL, which is lower than that of Trolox.

Trolox, a vitamin E analog, has the advantage of being moderately water soluble compared with vitamin E. Trolox has been widely used as a model compound of α-tocopherol [40]. Trolox, as a standard antioxidant compound, is applied for the expression of the antioxidant capacity of chemical compounds, food and biological matrices in terms of Trolox equivalent antioxidant capacity (TEAC) [41]. Hence, Trolox can be counted as a proper homolog of Vitamin E to investigate its behavior in aqueous radical environments [42]. The iron-reducing antioxidant capacity assay utilizes the ability of antioxidant polysaccharides to provide electrons for the reduction of Fe^3+^ to Fe^2+^. It is commonly used to evaluate the antioxidant capacity of polysaccharides [43]. The reducing capability of LEPS-1 increased in a dose-dependent manner in the range of 0.5–2.5 mg/mL (Figure 6A), and the concentration of Fe^2+^ reached 1.45 mM at 2.5 mg/mL. However, the reducing capability of LEPS-1 was still lower than that of Trolox in the range of 0.5–2.5 mg/mL. The reducing capability of LEPS-1 at 2.5 mg/mL was approximately equal to that of Trolox at 0.19 mg/mL. The DPPH free radical is commonly used as a representative reagent for assaying the radical scavenging activity of biologically active compounds. The antioxidant activity of polysaccharides is attributed to their hydrogen donor capacity [44]. Within the concentration range of 0.5–2.5 mg/mL, the DPPH radical scavenging capability increased with increased LEPS-1 concentration, and LEPS-1 was 13.95% at 2.5 mg/mL, which is lower than that of Trolox (Figure 6B).

#### 2.5.3. α-Amylase and α-Glucosidase Inhibitory Activity

**Figure 7:**
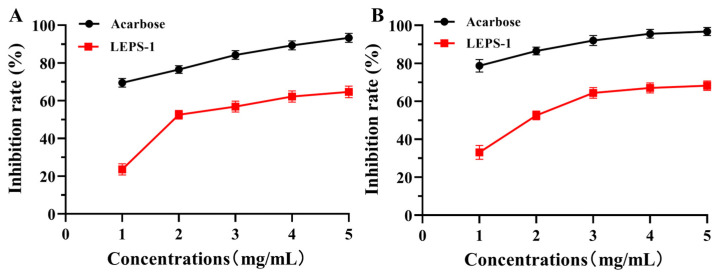
Effects of LEPS-1 on α-amylase (**A**) and α-glycosidase (**B**) inhibitory activities. The maximum inhibitory rates of LEPS-1 at 5.0 mg/mL on α-amylase and α-glucosidase were 64.220% and 68.220%, respectively.

Alpha amylase and α-glucosidase inhibitors may decrease glucose release from starch and delay carbohydrate absorption by inhibiting the activity of carbohydrate-hydrolyzing enzymes in the small intestine. Therefore, natural α-amylase and α-glycosidase inhibitors have attracted much attention [45]. LEPS-1 inhibited α-amylase activity and α-glucosidase activity in a dose-dependent manner within the test concentration (Figure 7). The maximum inhibitory rates of LEPS-1 on α-amylase and α-glucosidase were 64.220% and 68.220% at 5.0 mg/mL. Consequently, the corresponding IC_50_ values of LEPS-1 against α-amylase and α-glucosidase were 0.96 mg/mL and 1.92 mg/mL. However, the inhibition capacities of LEPS-1 on α-glucosidase and α-amylase were lower than that of the drug acarbose.

#### 2.5.4. Immunomodulatory Activity

**Figure 8:**
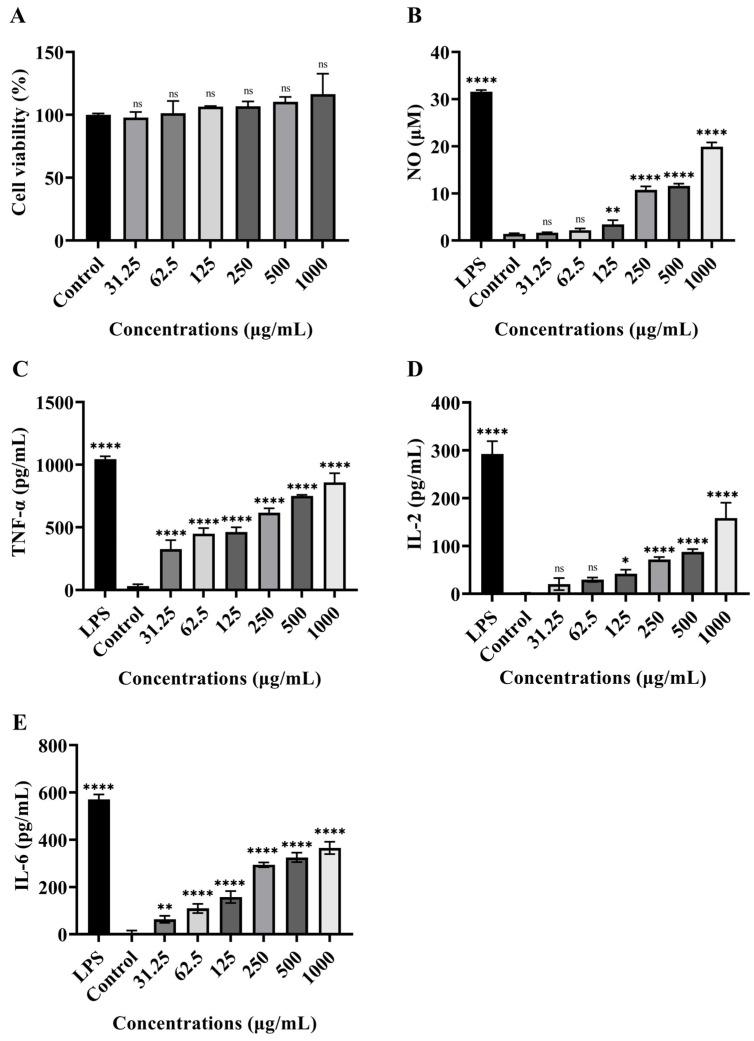
Effects of LEPS-1 on multiplication capacity of RAW 264.7 cells (**A**). Effects of LEPS-1 at different concentrations on production of NO (**B**), TNF-α (**C**), IL-2 (**D**) and IL-6 (**E**) in RAW 264.7 cells. The cells were pretreated with different concentrations of LEPS-1 for 24 h. The levels of NO, TNF-α, IL-2 and IL-6 in the supernatant were determined by ELISA. LEPS-1 presented no cytotoxicity at 31.25 to 1000 μg/mL. The NO production of RAW 264.7 cells treated with LEPS-1 at 1000 μg/mL was 19.90 μM. TNF-α production was 858.714 Pg/mL at 1000 μg/mL. LEPS-1 at 1000 μg/mL significantly stimulated IL-2 and IL-6 production in RAW 264.7 cells to 158.6 pg/mL and 365.1 pg/mL, respectively. Data show the mean ± SD of three independent experiments. The ns means no significance. * *p* < 0.05, ** *p* < 0.01 and **** *p* < 0.0001 compared with control group, n = 3.

The effect of LEPS-1 on RAW 264.7 cell proliferation was tested by the CCK-8 kit. As shown in Figure 8A, the proliferation of RAW 264.7 cells treated with LEPS-1 had no effect at 31.25 to 1000 μg/mL and did not exhibit any cytotoxicity. The production of NO in RAW 264.7 cells treated with LEPS-1 is shown in Figure 8B. As a positive control, LPS significantly stimulated NO production in RAW 264.7 cells to 31.55 μM. Within the range of 125–1000 μg/mL, LEPS-1 significantly increased NO production in a dose-dependent manner, while there were no effects at 31.25–62.5 μg/mL. The NO production of RAW 264.7 cells treated with LEPS-1 at 1000 μg/mL was 19.90 μM. After LPS stimulation, RAW 264.7 cells produce cytokines such as TNF-α, IL-2 and IL-6. As shown in Figure 8C, TNF-α production in the blank control group was 30.619 Pg/mL. At concentrations of 31.25–1000 μg/mL, LEPS-1 significantly promoted TNF-α production in a dose-dependent manner, and TNF-α production of RAW 264.7 cells exposed to LEPS-1 at 1000 μg/mL was 858.714 Pg/mL. Compared with the control group, LPS significantly stimulated IL-2 and IL-6 production in RAW 264.7 cells (Figure 8D). Interestingly, LEPS-1 significantly increased the concentrations of IL-2 (125–1000 μg/mL) and IL-6 (62.5–1000 μg/mL) in a dose-dependent manner. Treatment with LEPS-1 at 1000 μg/mL significantly stimulated IL-2 and IL-6 production in RAW 264.7 cells to 158.6 pg/mL and 365.1 pg/mL, respectively. Therefore, LEPS-1 significantly stimulated RAW 264.7 cells to release TNF-α, IL-2 and IL-6. Our results showed that LEPS-1 has no cytotoxic effect and stimulates RAW 264.7 cells to release NO, TNF, IL-2 and IL-6, showing greater potential for immunomodulatory activity.

## 3. Discussion

Recently, more and more researchers have turned their attention towards medicinal fungi, which are currently being evaluated for their nutritional value and acceptability as well as for their pharmacological and food properties [46]. Identification of the chemical structure of pure polysaccharides of mushrooms is paramount importance to the prospect of these products used in food or medicine [24]. This study reveals that the molecular weight of LEPS-1 was 36.131 kDa, the total sugar content was 86.48% and the proportion of uronic acid was 1.43%, which is a heteropolysaccharide mainly composed of mannose with a mole ratio of Fuc:Ara:Gal:Glc:Xyl:Man:Gal-UA:Gul-UA = 1.18:2.15:8.16:3.92:0.77:82.38:0.80:0.65. Structural characterization of LEPS-1 reveals that it is mainly composed of →2)-α-Man*p*(1→, →2,6)-α-Man*p*(1→, which forms the main chain with a small amount of →6)-α-Gal*p*(1→ and →3)-α-Man*p*(1→, and β-Man*p*(1→ mostly acts as a terminal sugar connecting to residue C at positions 6 and 2. Interestingly, polysaccharides with differing monosaccharide compositions differ in their biological activities. The antioxidant activity of polysaccharides is reduced with an increase in polysaccharide structural complexity and molecular weight. The uronic acid contained in the polysaccharide structure also has an important impact on antioxidant activity [18]. Ara 1→4 and Man 1→2 linkages of the branched chains in polysaccharides are positively correlated with their FRAP, whereas Glc 1→6 and Ara 1→4 linkages are related with their abilities of scavenging DPPH radicals [47]. A neutral mannan SSEPS2, composed of 1,3-linked and 1,2-linked α-D-mannopyranose (Manp), with a substitution at O-6 of 1, 2-linked α-D-Manp by 1,6-linked α-D-Manp residues and terminal α-D-Manp residues, demonstrated that it had potential antitumor activity on HepG2 and MCF-7 cells in vitro [23]. In some cases, polysaccharides with a high content/ratio of monosaccharides, such as Man, Rha and Fuc, appear to be responsible for the bioactivity [48]. Mannose is a typical component of many bioactive polysaccharides. Human cells are also able to recognize such carbohydrates through mannose receptors, thus stimulating cytokine production [49]. For example, Man-rich EPS from *Tremella mesenterica* [50] stimulate the immune system through receptors located on macrophages. The high molar proportion of mannose in *Inonotus obliquus* polysaccharides may enhance antioxidant activity [51,52]. Kim et al. reported an anti-cancer effect by IPS fractions from cultivated mycelia of *I. obliquus* with mannose as the major component [53]. In conclusion, it is reasonable to speculate that the biological activities of *S. sanghuang* JM-1 exopolysaccharides are closely related to their monosaccharide composition, molecular weight, glycosidic bond type and molar ratio and chain conformation, among other factors [54].

The antioxidant effects and hypoglycemic activity of polysaccharides have received extensive attention worldwide, and there is a growing desire to find natural and effective antioxidants and hypoglycemic substances [27]. Clinical studies reminded us that antioxidant-rich foods, such as *Phellinus* spp. (also called *Sanghuangporus* spp.), can slow down cellular aging and prevent the development of chronic diseases, including cancer, neurodegenerative diseases, cardiovascular diseases and diabetes [55]. Trolox, as a water-soluble vitamin E analog and a standard antioxidant compound, is applied for the expression of the antioxidant capacity of chemical compounds, food and biological matrices in terms of Trolox equivalent antioxidant capacity (TEAC) [41]. Radosław et al. [56] evaluated the antioxidant properties of aqueous and ethanolic extracts of the *Uncaria tomentosa* bark by Trolox. The reducing capability of LEPS-1 at 2.5 mg/mL was approximately 40.29% of that of Trolox. The DPPH radial scavenging ability of LEPS-1 at 2.5 mg/mL was 13.95%, and was approximately 24.87% of that of Trolox. Liu et al. [27] reported that the scavenging rates of the DPPH free radical exposed to five polysaccharides of *S. vaninii* at 1 mg/mL were 70.58 ± 0.72%, 56.73 ± 3.83%, 65.63 ± 2.17%, 61.84 ± 0.64 and 63.94 ± 0.98% for SVP-40, SVP-50, SVP-60, SVP-70 and SVP-80, which possessed stronger scavenging rates of the DPPH free radical than LEPS-1. As previous studies reported, the polysaccharides of phylogenetically related species of *S. sanghuang* exhibited excellent hypoglycemic activity [55]. Alpha amylase and α-glucosidase inhibitors may decrease glucose release from starch and delay carbohydrate absorption by inhibiting the activity of carbohydrate-hydrolyzing enzymes in the small intestine. Therefore, natural α-amylase and α-glycosidase inhibitors have attracted much attention [45]. The corresponding IC_50_ values of SSIPS1 (a polysaccharide isolated and purified from cultured mycelia of *S. sanghuang*) against α-amylase and α-glucosidase were 0.96 mg/mL and 0.91 mg/mL [10]. The corresponding IC_50_ values of LEPS-1 against α-amylase and α-glucosidase were 0.96 mg/mL and 1.92 mg/mL. Accordingly, this study showed that LEPS-1 isolated from *S. sanghuang* JM-1 exhibited α-amylase and α-glucosidase inhibitory activity. In addition, we will investigate the effects of LEPS-1 on glucose consumption in insulin-resistant HepG2 cells and the activities of hexokinase (HK) and pyruvate kinase (PK) in our next experiments. These findings further demonstrate that the in vivo biological activity of LEPS-1 needs to be further explored in terms of the detailed mechanisms [57].

Activated macrophages, which are a part of the innate immune system, are considered to be the pivotal immunocytes of host defense against tumor growth [58]. Macrophages can be activated to kill tumor cells by producing NO and TNF-α. Also, NO and TNF-α have been identified as the major effector molecules involved in the destruction of tumor cells by activated macrophages [59]. Moreover, PSs are the major active component in mushrooms and fungi, which can enhance innate and cell-mediated immune responses and which exhibit good immunomodulatory activity in animals and humans [60]. Polysaccharides can activate mouse macrophages through the TLR4 receptor, transmit signals, and activate downstream MAPK, nuclear factor (NF)-κB and other pathway proteins, mediating the occurrence of immune responses [61]. The immunomodulatory effects and mechanisms of two novel α-D-glucans (MIPB50-W and MIPB50-S-1) from *Morchella importuna* fruiting bodies were evaluated using murine RAW264.7 cells. MIPB50-W and MIPB50-S-1 can significantly promote the phagocytosis of FITC-dextran in RAW264.7 cells and the secretion of NO and TNF-α/IL-6, indicating that MIPB50-W and MIPB50-S-1 have immune activation activity in RAW264.7 cells [62]. *Phellinus baumii* polysaccharide increases the cellular proliferation rate, NO production, and expression levels of IL-1β, IL-18, IL-6, IL-12p35 and IL-12p40 genes in the RAW264.7 macrophages. We elucidated the regulatory role of LEPS-1 in the production of inflammatory cytokines in LPS-stimulated RAW 264.7 cells in vitro. Firstly, the effects of LEPS-1 on the viability of RAW 264.7 cells were studied, and the results showed that LEPS-1 presents no cytotoxicity. Our study found that LEPS-1 stimulates RAW 264.7 cells to release NO, TNF-α, IL2 and IL6, showing potential for immunomodulatory activity.

Recent studies have shown that *S. sanghuang* polysaccharides (SSPs) can enhance immunity and anti-cancer effects without obvious toxicity towards target organs. However, the structure of SSP is complex, and the relationship between its structure characteristics and activity is unclear, limiting its application [15]. Our findings also provide considerably valuable clues and new insights that elaborate the structure–bioactivity relationships of SSP. SSP can also be made into final products by itself or in combination with other functional ingredients in the form of dried slices, powders, tablets, tea drinks, extract pastes, etc., and is widely used in functional food, versatile medicine and cosmetics. Moreover, the literature proposed that the solubility of polysaccharides reflects their application value. In this study, LEPS-1 was easily soluble in water, which indicates a higher hydrophilicity and better water retention. Therefore, various pieces of evidence have proved that PSs with lower molecular weight have higher antioxidant activity and immunomodulatory activity, and often have better solubility [62,63,64]. Given the above results, it would be interesting to investigate other bioactivities and physical properties of LEPS-1, to alter the LEPS-1 molecular structure to produce new effects with chemical modifications such as sulfation and selenization [65]. 

## 4. Materials and Methods

### 4.1. Materials and Chemicals

The *S. sanghuang* strain JM-1 (Figure S1) was preserved at the Institute of Microbiology Research, Guangxi Academy of Agricultural Sciences (Nanning, China). The NCBI accession number is OR363177.

In this study, all materials were commercially available. Sephadex G-75 and the CCK-8 kit were provided by Solarbio (Beijing, China). The 25% Trypsin-EDTA, the BCA kit, α-amylase and potassium bromide were obtained from Aladdin Biochemical Technology (Shanghai, China). The Mouse ELISA kit for TNF-α, IL-2 and IL-6 was purchased from Enzyme-linked Biotechnology Co., Ltd. (Shanghai, China). 

### 4.2. Extraction and Purification of EPS

Fermentation medium (corn meal 5.4 g/L, glucose 17.0 g/L, peptone 5.8 g/L, VB_1_ 10.0 mg/L, KH_2_PO_4_ 3.0 g/L, MgSO_4_·7H_2_O 5.0 g/L) was used to culture *S. sanghuang* JM-1. The cultures were filtered to collect the fermentation broth. Then 3-fold volumes of anhydrous ethanol were added into the obtained fermentation broth to precipitate the crude exopolysaccharides for 24 h at 4 °C. After being centrifuged at 8000 rpm for 20 min, the precipitate was collected and deproteinized with the Sevage method [21]. After that, the resulting supernatant was dialyzed with a dialysis bag (Mw 3.5kD, MD 55 mm) and lyophilized to obtain a crude EPS (named LEPS). The pigment of LEPS was removed by D101 macroporous adsorption resin. The decolorized LEPS was purified on a DEAE-52 column and Sephadex G-75 gel column. The obtained fraction of LEPS was dialyzed with a dialysis bag (Mw 3.5 kD, MD 55 mm) and lyophilized to obtain a purified EPS (named LEPS-1). 

### 4.3. Chemical Composition Analysis of LEPS-1

The LEPS-1 was dissolved in distilled water to prepare a 1 mg/mL solution for scanning UV spectra (EVOLΜTION, Thermo Fisher Scientific, Waltham, MA, USA) in the range of 200 to 500 nm. The contents of total sugars, protein and uronic acids in LEPS-1 were determined by the phenol–sulfuric acid method with glucose as a standard, the bicinchoninic acid (BCA) method, and m-hydroxybiphenyl methods, respectively [18].

### 4.4. Determination of Monosaccharide Composition of LEPS-1

The thin layer chromatography (TLC) was performed as described previously [66], with some modification to analyze the monosaccharide composition of LEPS-1. In brief, eight monosaccharide standards were dissolved in distilled water to prepare a 1 mg/mL solution. The LEPS-1 (5 mg) was hydrolyzed using 2 mL of 2 M trifluoroacetic acid (TFA) at 115 °C for 6 h. After that, we removed water and TFA from the sample and then added methanol to the dry sample and evaporated it under reduced pressure, and finally added 5 mL of distilled water to dissolve it. For the TLC assay, standards and hydrolysate of LEPS-1 were released in a N-butanol:pyridine:water:ammonia (8:4:1.5:0.025, V/V) mobile phase using TLC plates (Merck, Darmstadt, Germany). After drying TLC plates, the samples were colorized with Licharl sulfuric acid solution at 105 °C for 15 min.

Referring to the methods reported by Zhang et al. [39] with minor modifications, on the basis of monosaccharide standards in TLC, ribose, galacturonic acid, glucuronic acid, mannuronic acid and guluronic acid were added to prepare a standard solution with final concentrations of 0.5, 1, 5, 10, 20, 25, 30, 40, and 50 μg/mL. The above hydrolysate of LEPS-1 was determined using the Thermo ICS 5000 ion chromatography system (ICS5000, Thermo Fisher Scientific, Waltham, MA, USA) equipped with a liquid chromatography column Dionex™ CarboPac™ PA20 (150 × 3.0 mm, 10 μm) to analyze monosaccharide composition. The detection conditions were as follows: the injection volume was 5 μL; phase A was 0.1 M NaOH and phase B was 0.1 M NaOH, 0.2 M NaAc with a flow rate of 0.5 mL/min; column temperature, 30 °C; and the elution gradient: 0 min A/B (95:5, V/V), 30 min A/B (80:20, V/V), 30.1 min A/B (60:40, V/V), 45 min A/B (60:40, V/V), 45.1 min A/B (95:5, V/V) and 60 min A/B (95:5, V/V).

### 4.5. Molecular Weight Determination of LEPS-1

The molecular weight determination was performed as described previously [36], with some modification. Briefly, the detection conditions were as follows: sample concentration was 1 mg/mL; column temperature, 45 °C; mobile phase 0.1 M NaNO_3_; injection volume was 100 μL; and the flow rate was 0.5 mL/min. 

### 4.6. Characterization of Chemical Structure of LEPS-1

#### 4.6.1. Methylation and Gas Chromatography-Mass Spectrometry Analysis

The chemical structure of LEPS-1 was determined with methylation using a modified method reported by Liu et al. [19]. LEPS-1 (10 mg) was completely dissolved in 500 μL dimethyl sulfoxide (DMSO). After adding NaOH (10 mg), the mixture was ultrasonicated for 30 min. After that, 50 μL iodomethane (CH_3_I) was added to the resulting mixture and maintained for 60 min. Then, 1 mL water was added to end the reaction. The dichloromethane (2 mL) was mixed with the resulting mixture and centrifuged to collect the lower layer of dichloromethane phase. The mixture was hydrolyzed using 2 M TFA (100 μL) at 100 °C for 90 min and evaporated at reduced pressure. After adding ammonia and NaBH_4_ (50 μL) at room temperature for 3 h, the reaction was stopped by adding acetic acid (20 μL). Methanol (250 μL) was added to the mixture and maintained at 100 °C for 2 h. The final product was dissolved in dichloromethane (1 mL) and filtered with 0.45 µm nylon filter, and then analyzed by GC-MS (Agilent, Santa Clara, CA, USA*,* 7890A-5977B) with reference to the method reported by Zhang et al. [36].

#### 4.6.2. Fourier Transform Infrared (FT-IR) Spectroscopy Measurement

FT-IR spectroscopy of LEPS-1 was performed as described by Liu et al. [19]. A total of 2 mg LEPS-1 was mixed with 200 mg potassium bromide and pressed into sample tablets, followed by Nicolet iZ-10 (Thermo Fisher Scientific, USA) analysis in the range of 4000~400 cm^−1^ with a resolution of 4 cm^−1^ and 32 scans.

#### 4.6.3. Nuclear Magnetic Resonance (NMR) Spectroscopy Measurement

LEPS-1 was dissolved in D_2_O at 20 mg/mL and added into the nuclear magnetic tube. ^1^H NMR and ^13^C NMR spectroscopy, ^1^H-^1^H correlation spectroscopy (COSY), heteronuclear single-quantum coherence (HSQC), heteronuclear multiple-bond correlation (HMBC) and nuclear Overhauser effect spectroscopy (NOESY) of the polysaccharide solution were performed on the NMR spectrometer (AV-500 Bruker BioSpin GmbH, Ettlingen, Germany) at 333.15 K.

### 4.7. Bioactivity Analysis of LEPS-1

#### 4.7.1. Solubility

The solubility of the sample was determined by the weight balance method with minor modification [67]. The LEPS-1 was collected with 10 mg and added into 1 mL distilled water, standing at room temperature for 90 min. Then, it was vortexed for 5 s at 8000 r/min and underwent centrifugation for 30 min at 20 °C. The solubility formula is as follows:

Solubility (%) = (W_1_ − W_2_)/W_0_ × 100%

where W_1_ is the mass of sample and tube, mg; W_2_ is the mass of precipitation and tube, mg; and W_0_ is the mass of sample, mg.

#### 4.7.2. Antioxidant Activity In Vitro

Reduction capacity was determined according to the instructions for the FRAP kit.

According to the method of Zheng et al. [68] DPPH radical clearance ability was determined. Trolox was used as a positive control. In short, LEPS-1 and Trolox were dissolved in distilled water to form different concentrations. The solutions (2 mL) were added to 0.2 mmol/L DPPH anhydrous ethanol solution (2 mL). After mixing, the absorbance was measured at 517 nm in the dark at room temperature for 10 min. The absolute ethanol solution was used as a blank, and the DPPH radical clearance were calculated using the formula given in Equation (1):

(1) $$\mathrm{the}\;\mathrm{DPPH}\;\mathrm{radical}\;\mathrm{clearance}\;(\%)=1-\frac{A1-A2}{A3}\times 100\%$$

In this formula, A_1_ is absorbance of sample solution (2 mL) + DPPH-anhydrous ethanol solution (2 mL); A_2_ is absorbance of sample solution (2 mL) + anhydrous ethanol (2 mL); and A_3_ is absorbance of DPPH-anhydrous ethanol solution (2 mL) + anhydrous ethanol (2 mL).

#### 4.7.3. α-Amylase and α-Glucosidase Inhibitory Activity In Vitro

α-Amylase inhibitory activity was determined using the reported methods [69]. The inhibition test of α-amylase activity was determined. The acarbose was used as a positive control. Briefly, LEPS-1 and acarbose were dissolved in 0.1 mol/L pH 6.8 phosphate buffer to form different concentrations. The solutions (1 mL) were added to 1 unit/mL α-amylase solution (1 mL) in 37 °C water for 10 min. The mixture was added to 0.25% (*w*/*v*) starch solution (1 mL) at 37 °C for 10 min. Then the above solution was added to DNS solution (1 mL), and reacted in boiling water for 5 min. After that, the above reacted solution rapidly moved to ice water to cool it. Then the absorbance was measured at OD_540_. The phosphate buffer solution was used as a blank, and the inhibition rate of α-amylase was calculated using the formula given in Equation (2):

(2) $$\mathrm{The}\;\mathrm{inhibition}\;\mathrm{rate}\;\mathrm{of}\;\alpha -\mathrm{amylase}\;(\%)=1-\frac{A1-A2}{A3-A4}\times 100\%$$

In the formula, A_1_ is absorbance of sample solution (1 mL) + α-amylase solution (1 mL) + 0.25% starch solution (1 mL) + DNS solution (1 mL); A_2_ is the absorbance of sample solution (1 mL) + phosphate buffer (1 mL) + 0.25% starch solution (1 mL) + DNS solution (1 mL); A_3_ is absorbance of phosphate buffer (1 mL) + 0.25% starch solution (1 mL) + DNS solution (1 mL); and A_4_ is absorbance of phosphate buffer (1 mL) + 0.25% starch solution (1 mL) + DNS solution (1 mL).

The α-glucosidase activity inhibition test was determined according to the instructions for the kit.

#### 4.7.4. Immunomodulatory Assays

##### Cytotoxicity Assay

LEPS-1 was determined by the CCK-8 assay (Solarbio, Beijing, China). The density of mouse RAW 264.7 cells was adjusted to 1 × 10^7^ Cfu/mL and cultured in 96-well plates at 100 μL per well. After the cells were stable, polysaccharide solution (10 μL) of different concentrations was added, with the final concentrations of 31.25, 62.5, 125, 250, 500 and 1000 μg/mL. Using sterile water as a blank control, CCK-8 solution (10 μL) was added to each well for 1 h. The solution absorbance was measured with a microplate reader.

##### Quantification of NO, TNF-α, IL-2 and IL-6

The density of mouse RAW 264.7 cells was adjusted to 1 × 10^7^ Cfu/mL and cultured in 96-well plates at 100 μL per well. After the cells were stable, LEPS-1 solution (10 μL) of different concentrations was added, with the final concentrations of 31.25, 62.5, 125, 250, 500, 1000 μg/mL. Sterile water was used as a blank control and LPS (1 μg/mL) was used as a positive control. After further cultivation for 24 h, the cell supernatant was determined for NO, TNF-α, IL-2 and IL-6 with the NO kit and the Mouse ELISA kit.

### 4.8. Statistical Analyses

Data processing and analysis were conducted using GraphPad Prism 9.0, Origin 2021, and SPSS.24. Statistical significance was determined by Dunnett’s multiple comparisons tests. Values are expressed as means ± SD. * *p* < 0.05, ** *p* < 0.01 and **** *p* < 0.0001 were considered statistically significant.

## 5. Conclusions

LEPS-1 is an exopolysaccharide purified from *S. sanghuang* JM1 which is composed of Fuc:Ara:Gal:Glc:Xyl:Man:Gal-UA:Gul-UA with a molar ratio of 1.18:2.15:8.16:3.92:0.77:82.38:0.80:0.6, and mannose was the predominant monosaccharide in LEPS-1. LEPS-1 contained a →2)-α-Manp(1→6)-α-Galp(1→ [2)-α-Manp(1→]_n_→2,6)-α-Manp(1→6,2)-α-Manp(1→3)-α-Manp(1→ backbone substituted at the *O*-6 and *O*-2 positions with side chains. These two branching fragments were β-Manp(1→. The molecular weight of LEPS-1 is 36.131 kDa. LEPS-1 was easily soluble in water with reducing capability and DPPH radical scavenging capability. Further, the IC_50_ values of LEPS-1 against α-amylase and α-glucosidase were 0.96 mg/mL and 1.92 mg/mL. LEPS-1 could stimulate RAW264.7 cells to release NO, TNF-α, IL-2 and IL-6 with no cytotoxicity, showing potential for immunomodulation activity. These findings describe a potential natural exopolysaccharide with medicinal value and a basis for the development of *S. sanghuang* exopolysaccharides.

## Figures and Tables

**Table 1:** Methylation analysis of LEPS-1.

Connection Mode	Derivative Name	Molecular Weight (M_W_)	Relative Molar Ratio (%)
t-Ara(*f*)	1,4-di-O-acetyl-2,3,5-tri-O-methyl arabinitol	279	1.970
t-Fuc(*p*)	1,5-di-O-acetyl-6-deoxy-2,3,4-tri-O-methyl fucitol	293	0.61
t-Man(*p*)	1,5-di-O-acetyl-2,3,4,6-tetra-O-methyl mannitol	323	35.70
3-Man(*p*)	1,3,5-tri-O-acetyl-2,4,6-tri-O-methyl mannitol	351	5.07
2-Man(*p*)	1,2,5-tri-O-acetyl-3,4,6-tri-O-methyl mannitol	351	24.41
6-Glc(*p*)	1,5,6-tri-O-acetyl-2,3,4-tri-O-methyl glucitol	351	3.91
4-Glc(*p*)	1,4,5-tri-O-acetyl-2,3,6-tri-O-methyl glucitol	351	0.82
6-Gal(*p*)	1,5,6-tri-O-acetyl-2,3,4-tri-O-methyl galactitol	351	7.17
3,6-Man(*p*)	1,3,5,6-tetra-O-acetyl-2,4-di-O-methyl mannitol	379	0.82
2,6-Man(*p*)	1,2,5,6-tetra-O-acetyl-3,4-di-O-methyl mannitol	379	18.07
3,6-Gal(*p*)	1,3,5,6-tetra-O-acetyl-2,4-di-O-methyl galactitol	379	0.31
2,6-Gal(*p*)	1,2,5,6-tetra-O-acetyl-3,4-di-O-methyl galactitol	379	1.14

**Table 2:** ^1^H and ^13^C chemical shifts of the LEPS-1.

Glycosyl Residues	Chemical Shifts (ppm)					
	H1/C1	H2/C2	H3/C3	H4/C4	H5/C5	H6/C6
A β-Manp(1→	4.97	4.14	3.96	3.68	3.58	3.82
	102.21	69.66	78.67	73.40	66.91	61.14
B →2)-α-Manp(1→	5.22	4.04	3.67	3.69	3.55	3.68
	100.57	78.51	66.69	73.42	67.02	61.15
C →2,6)-α-Manp(1→	5.06	3.95	3.74	3.70	3.75	3.82
	102.34	78.76	66.71	73.36	73.42	70.47
D →6)-α-Galp(1→	5.02	3.95	3.81	3.71	3.48	3.99
	98.26	65.93	78.87	66.35	78.87	70.18
E →3)-α-Manp(1→	4.91	3.80	3.87	3.58	3.47	3.84
	97.89	67.22	77.97	73.36	78.76	61.27

## Data Availability

The data presented in this study are available on request from the corresponding authors.

## Supplementary Materials

The following supporting information can be downloaded at: https://www.mdpi.com/article/10.3390/molecules29194564/s1.
