# Supplementary material for: Structural Characterization, and Antioxidant, Hypoglycemic and Immunomodulatory Activity of Exopolysaccharide from Sanghuangporus sanghuang JM-1
Source: Molecules. 2024 Sep 25;29(19):4564. doi: 10.3390/molecules29194564 (PMC11477767; doi:10.3390/molecules29194564)
Supplement: Supplementary file 1 [file molecules-29-04564-s001.zip › molecules-3202559-supplementary.pdf]

Tables:

Table S1 The retention time and amount of monosaccharides in standard and LEPS-1

Peak Name	Retention Time (min)		Amount (%)	
	standard	LEPS-1	standard	LEPS-1
Fuc	3.667	3.667	8.8213	0.5128
Ara	7.717	7.725	8.9286	0.9351
Rha	8.042	n.a.	8.6390	n.a.
Gal	9.517	9.559	8.7172	3.5482
Glc	11.259	11.292	9.4602	1.7051
Xyl	13.425	13.467	9.1175	0.3344
Man	14.600	14.634	10.3451	35.8347
Fru	16.359	n.a.	11.5045	n.a.
Rib	17.509	n.a.	12.1202	n.a.
Gal-UA	34.642	34.659	11.8554	0.3461
Gul-UA	35.225	35.225	11.5446	0.2812
Glc-UA	37.467	n.a.	12.1753	n.a.
Man-UA	40.250	n.a.	10.7026	n.a.

Figures:

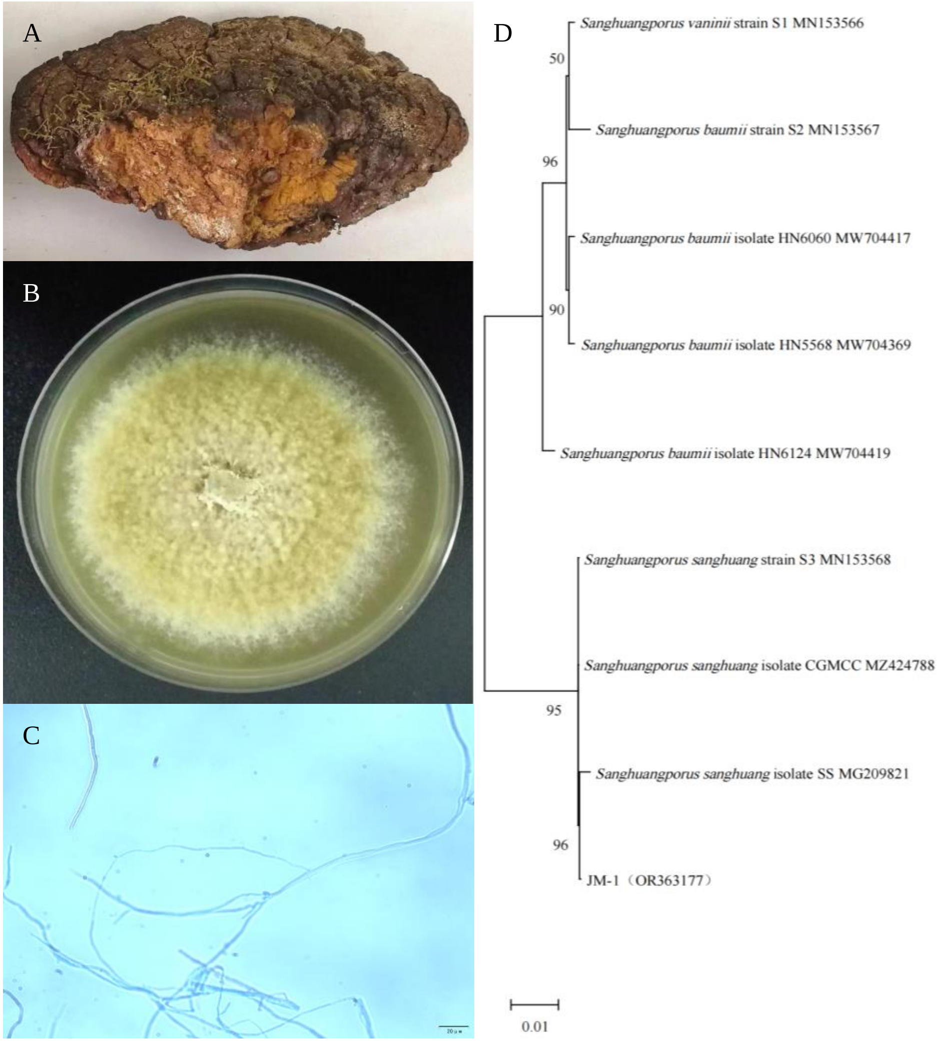

Figure S1. The fruiting body (A), colonial morphology (B), microscopic appearance of hyphae (C) and phylogenetic tree (D) of *S. sanghuang* JM-1.

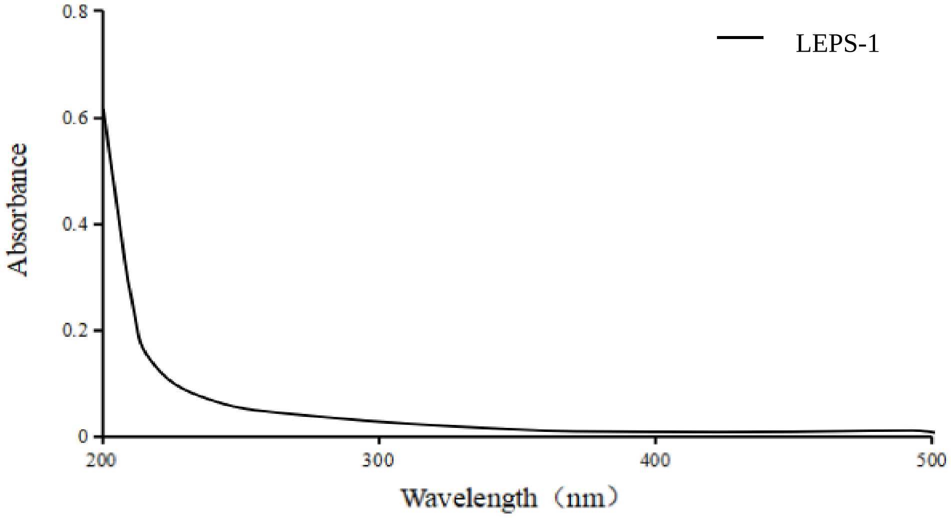

Figure S2. UV spectrum of LEPS-1.

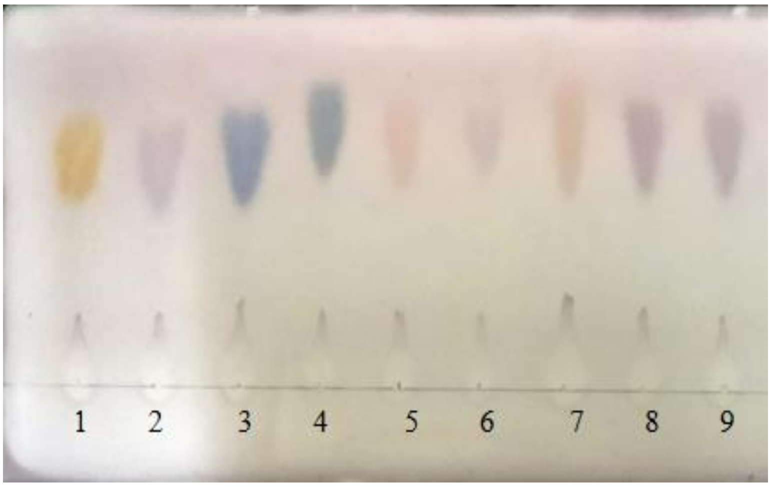

Figure S3. TLC of standard monosaccharides and hydrolysate of LEPS-1 (1, Fructose; 2, Galactose; 3, Glucose; 4, Arabinose; 5, Fucose; 6, Xylose; 7, Rhamnose; 8, Mannose; 9, Hydrolysate of LEPS-1).

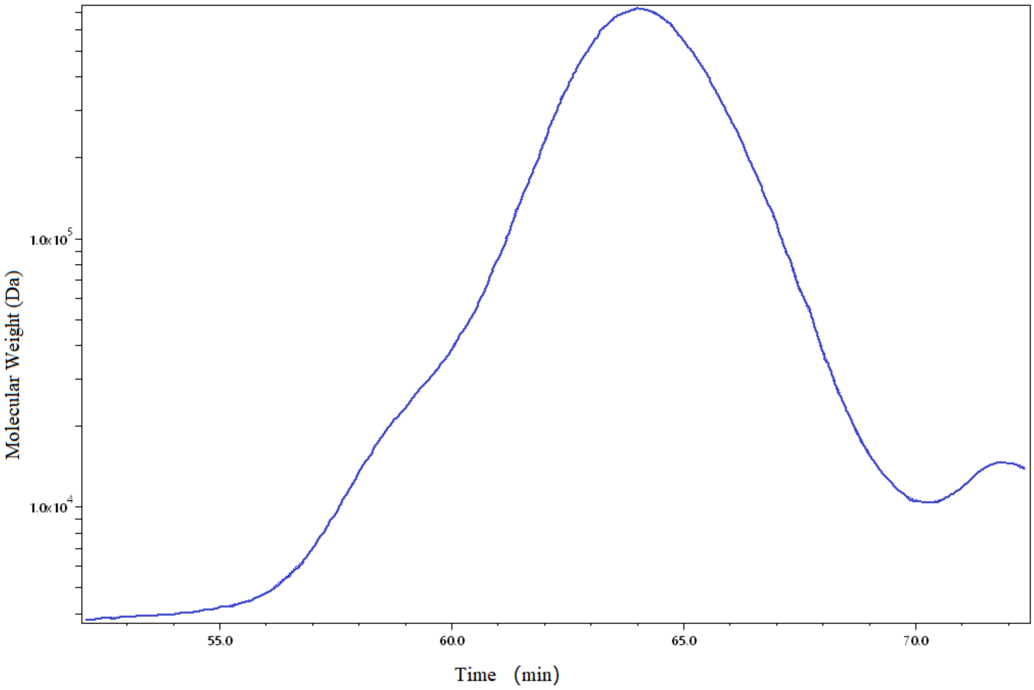

Figure S4. HPSEC chromatogram of LEPS-1.

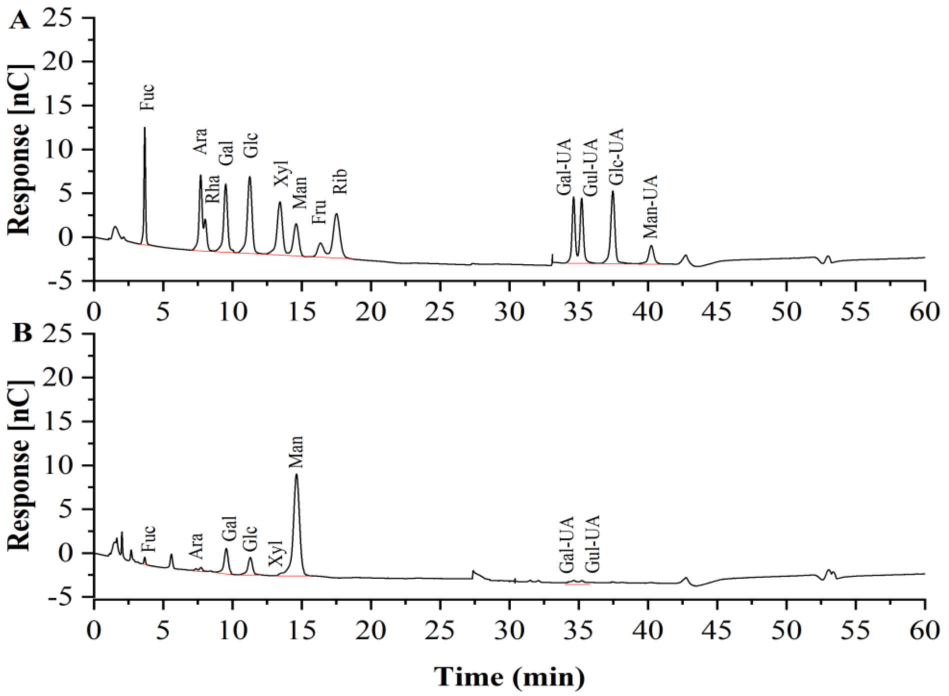

Figure S5. HPLC chromatograms of standard monosaccharides (A) and LEPS-1 (B).

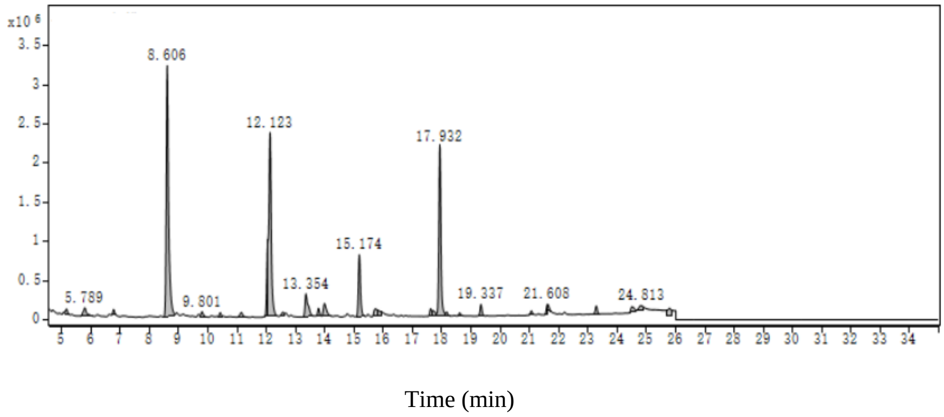

Figure S6.Total ion chromatogram(TIC) of LEPS-1
